# IFN-γ–dominant immune shift is associated with reduced protective ILC2 responses in chronic kidney disease

**DOI:** 10.3389/fimmu.2026.1870937

**Published:** 2026-09-08

**Authors:** Yoshihiro Kuno, Hiroki Ishikawa, Ryuichi Nagashima, Yasunari Matsuzaka, Chikara Kohda, Nobuhiro Kanazawa, Taihei Suzuki, Takeo Isozaki, Hirotaka Kuwata, Masayuki Iyoda

**Affiliations:** 1 Department of Microbiology and Immunology, Showa Medical University Graduate School of Medicine, Tokyo, Japan; 2 Division of Nephrology, Department of Medicine, Showa Medical University Graduate School of Medicine, Tokyo, Japan; 3 Division of Immunology, Department of Biosciences, Kitasato University School of Science, Sagamihara, Japan; 4 Department of Pathogenesis and Translational Medicine, Showa Medical University Graduate School of Pharmacy, Tokyo, Japan; 5 Department of Oral Microbiology and Immunology, Showa Medical University Graduate School of Dentistry, Tokyo, Japan

**Keywords:** CKD, fibrosis, IFN-γ, ILC2, NKT cells

## Abstract

Chronic kidney disease (CKD) progression is driven by an imbalance between inflammatory injury and tissue repair; however, the immune mechanisms that disrupt reparative pathways remain incompletely understood. Group 2 innate lymphoid cells (ILC2s) play protective roles in epithelial integrity and antifibrotic responses in the kidney, yet the signals that limit their maintenance during chronic inflammation are unclear. In this study, we investigated whether interferon-γ (IFN-γ)–dominant immune polarization is associated with the loss of renal ILC2s and exacerbation of CKD. Using a murine model of adenine-induced CKD, administration of α-galactosylceramide (αGalCer), a glycolipid known to activate invariant NKT cells, exacerbated renal dysfunction and tubulointerstitial injury. This was accompanied by enhanced inflammatory responses and a shift toward a type 1–dominant immune milieu. Notably, αGalCer treatment was associated with reduced renal ILC2 and regulatory T cell abundance, along with suppression of type 2 cytokines. Time-course analysis revealed a rapid and transient increase in renal *Ifng* expression following αGalCer administration, suggesting an early cytokine-driven immune shift. Neutralization of IFN-γ ameliorated kidney injury, restored ILC2 abundance, and partially rebalanced the renal immune environment. These findings suggest that IFN-γ–dominant immune polarization is associated with suppression of protective ILC2 responses and exacerbation of CKD. Further investigation of IFN-γ–mediated immune imbalance may provide insights into therapeutic strategies aimed at preserving protective ILC2 responses in CKD.

## Introduction

Chronic kidney disease (CKD) affects more than 800 million people worldwide and remains a major cause of morbidity and mortality (1). CKD progression is characterized by persistent tubular injury, interstitial inflammation, and dysregulated repair processes that ultimately lead to tubulointerstitial fibrosis and irreversible nephron loss (2). Although fibrosis represents the final common pathway of CKD, the upstream immune mechanisms that disrupt tissue repair and drive this transition remain incompletely understood.

Group 2 innate lymphoid cells (ILC2s) have emerged as key tissue-resident regulators of epithelial protection and repair in the kidney (3, 4). Renal ILC2s respond to epithelial-derived alarmins such as IL-33 and produce amphiregulin and type 2 cytokines that support epithelial integrity, limit inflammation, and restrain fibrotic remodeling (3, 4). Experimental studies have demonstrated that expansion of renal ILC2s ameliorates kidney injury and attenuates fibrosis in multiple models, including unilateral ureteral obstruction and adenine-induced CKD (5, 6). Despite these protective roles, ILC2s are markedly reduced during chronic kidney injury (5, 6), suggesting that active suppressive signals limit their reparative capacity.

Interferon-γ (IFN-γ) is a central mediator of type 1 immune responses and a potent negative regulator of ILC2 differentiation and function (7, 8). Increased IFN-γ signaling promotes type 1 immune polarization and suppresses type 2 immunity, thereby disrupting tissue repair processes in various organs (9, 10). However, the regulatory context and upstream triggers of IFN-γ–dominant immune environments that contribute to ILC2 suppression in the kidney remain unclear.

Natural killer T (NKT) cells are innate-like lymphocytes capable of rapidly producing large amounts of cytokines, including IFN-γ, upon activation (11). NKT cells are present in the kidney and respond early to tissue injury (12–14), and previous studies have demonstrated that they modulate renal inflammation and fibrosis in a context-dependent manner (15–19). These observations raise the possibility that excessive or dysregulated immune activation leading to increased IFN-γ production may contribute to suppression of reparative pathways in CKD.

In this study, we examined whether IFN-γ–dominant immune polarization is associated with loss of renal ILC2s and exacerbation of CKD. The adenine-induced CKD model is a well-established experimental model characterized by crystal-associated tubulointerstitial injury, inflammation, and progressive fibrosis, and is widely used to investigate the mechanisms underlying CKD progression (20). Using a murine model of adenine-induced CKD, we evaluated the effects of α-galactosylceramide (αGalCer) administration on renal injury, immune composition, and cytokine responses. αGalCer is a synthetic glycolipid that potently activates invariant natural killer T (iNKT) cells through CD1d-mediated antigen presentation, resulting in rapid production of cytokines including IFN-γ and broad modulation of innate and adaptive immune responses (11). We further assessed the contribution of IFN-γ using *in vivo* neutralization approaches.

Our findings suggest that an IFN-γ–dominant immune environment is associated with suppression of renal ILC2s and impaired tissue repair, thereby contributing to CKD progression. These results highlight immune imbalance between type 1 and type 2 responses as a potential driver of disease progression and suggest therapeutic strategies aimed at preserving ILC2-mediated renal protection.

## Materials and methods

### Experimental protocol

Chronic kidney injury was induced by feeding mice the adenine-containing diet for 2 wks. Control mice received the MF control diet (Oriental Yeast Co., Ltd., Tokyo, Japan). At the onset of adenine feeding (day 0), mice were intraperitoneally injected with either vehicle (2.5% DMSO in PBS) or αGalCer (Funakoshi Co., Ltd., Tokyo, Japan; Lot. 21F-16A) at 2 μg or 5 μg per mouse. αGalCer was dissolved in DMSO at a concentration of 1 mg/mL and stored at −20 °C. Immediately before administration, the stock solution was diluted with PBS to the indicated dose, with the final DMSO concentration adjusted to approximately 2.5%. The final injection volume was 200 μL per mouse.

The doses of αGalCer were selected based on our preliminary dose-comparison experiment. An initial comparison of 2 μg and 5 μg demonstrated that the 5 μg dose induced a more robust biological response and was therefore used in all subsequent experiments.

For dose-comparison experiments, mice were assigned to the following groups: control diet, adenine + vehicle, adenine + αGalCer (2 μg), and adenine + αGalCer (5 μg). For time-course experiments, mice were fed either a control or adenine-containing diet, administered αGalCer (5 μg), and euthanized on days 1, 3, and 5 after treatment for analysis of early gene expression changes. For IFN-γ neutralization experiments, adenine-fed mice treated with αGalCer (5 μg) received intravenous injections of anti–IFN-γ monoclonal antibody (clone XMG1.2; Lot. D0406-P079J, SouthernBiotech, Birmingham, AL, USA) at 50 μg per mouse on days −3, 0, 3, and 6. Control mice received the same dose of isotype control antibody (rat IgG1; Abcam, Cambridge, UK). Unless otherwise indicated, all remaining experiments were performed using adenine-fed mice treated with vehicle or αGalCer (5 μg), and tissues and blood were collected on day 14.

Sample sizes were determined on the basis of our previous studies using the adenine-induced CKD model (5), in which comparable group sizes were sufficient to detect biologically relevant differences. Each individual mouse represented one biological replicate. Animals were randomly assigned to experimental groups, and no animals were excluded from the analyses. Mice were euthanized by gradual-fill carbon dioxide inhalation at a flow rate of approximately 30% of the chamber volume per minute.

### BUN and creatinine determination

Two wks after initiation of diet feeding, mice were euthanized by CO_2_ inhalation. Blood was collected via cardiac puncture and centrifuged to obtain serum. BUN and creatinine levels in the serum were measured using the urease-GLDH method and an enzymatic method, respectively, by Oriental Yeast Co., Ltd. In some experiments, both kidneys were harvested from each mouse after blood collection for further analysis.

### Purification of mRNA from kidney and quantitative real-time PCR

Kidneys removed from mice were homogenized in a bead-based tissue homogenizer using MagNA Lyser Green Beads (Roche Diagnostics, Mannheim, Germany) and a Micro Smash™ tissue homogenizer (TOMY Seiko Co. Ltd., Tokyo, Japan). Total RNA from kidney was purified using RNeasy Mini kit^®^ (Qiagen, Hilden, Germany), and cDNA was synthesized using QuantiTect^®^ Reverse Transcription (Qiagen) according to the manufacturer’s instructions. The primers used for quantitative real-time PCR in this study were synthesized by Eurofins Genomics Japan (Tokyo, Japan) (**Table 1**). Quantitative real-time PCR was performed using SYBR™ Green qPCR Master Mix (ThermoFisherScientific, MA, USA) and QuantStudio 3 real-time PCR system with associated software (ThermoFisherScientific). Each sample was calibrated to *Hprt1* as an internal control and normalized to the average value of samples using the ΔΔCT method.

**Table 1:** Primer sequences for quantitative RT-PCR.

Gene name	Forward primer (5’-3’)	Reverse primer (5’-3’)
*Acta2*	TCGGATACTTCAGCGTCAGGA	GTCCCAGACATCAGGGAGTAA
*Col1a1*	AGACATGTTCAGCTTTGTGGAC	GCAGCTGACTTCAGGGATG
*Tgfb1*	CTGCTGACCCCCACTGATAC	AGCCCTGTATTCCGTCTCCT
*Fn1*	TGGTGGCCACTAAATACGAA	GGAGGGCTAACATTCTCCAG
*Il1b*	AGTTGACGGACCCCAAAAG	AGCTGGATGCTCTCATCAGG
*Il6*	CCTTCTTGGGACTGATGCTGGT	GACAGGTCTGGTTGGGAGTGGTAT
*Tnfa*	CGTCAGCCGATTTGCTATCT	CGGACTCCGCAAAGTCTAAG
*Lcn2*	ACGGACTACAACCAGTTCGC	AATGCATTGGTCGGTGGGG
*Havcr1*	TCCACACATGTACCAACATCAA	GTCACAGTGCCATTCCAGTC
*Ifng*	GAACTGGCAAAAGGATGGTGA	TGTGGGTTGTTGACCTCAAAC
*Il33*	GGCTGCATGCCAACGACAAGG	AAGGCCTGTTCCGGAGGCGA
*Il4*	ATCATCGGCATTTTGAACGAGGTC	ACCTTGGAAGCCCTACAGACGA
*Il5*	TGACAAGCAATGAGACGATGAGG	ACCCCCACGGACAGTTTGATTC
*Il13*	ACAAGACCAGACTCCCCTGT	TCTGGGTCCTGTAGATGGCA
*Hprt1*	TCCTCCTCAGACCGCTTTT	CCTGGTTCATCATCGCTAATC

### Histopathological evaluation of tubulointerstitial injury

Excised kidneys were fixed in 10% neutral-buffered formalin (Fujifilm, Tokyo, Japan) and processed for histological analysis. Briefly, fixed tissues were sectioned at a thickness of 4 μm using a microtome and stained with Masson’s trichrome by Soshiki Kagaku Lab Inc. (Kanagawa, Japan). To assess tubulointerstitial injury, three representative fields, corresponding to the upper, middle, and lower poles of the cortex, were selected from each stained section and evaluated at ×100 magnification using ImageJ software (NIH, MD, USA). Tubulointerstitial damage was quantified as the percentage of cortical area exhibiting interstitial tissue remodeling and tubular dilatation in each field. The average of these three fields was used as the final score for each sample. All histological evaluations were performed in a blinded manner by two independent investigators, and the mean of their assessments was calculated.

### Flow cytometry analysis

Flow cytometry analysis of cell types in the kidney was performed according to previously described methods (5). Briefly, kidneys were removed, minced, and digested in RPMI 1640 containing 0.5 mg/ml collagenase type I (Fujifilm, Osaka, Japan), 0.5 mg/ml dispase II (Roche, Basel, Switzerland) and 50 μg/ml DNase I (Roche) at 37 °C for 30 min. The dissociated kidney tissue was homogenized and centrifuged. The pellet was suspended in 40% Percoll (Cytiva, Marlborough, MA, USA), overlaid on 80% Percoll, and then centrifuged at 1000 ×*g* for 20 min. The interface layer was collected as lymphocytes, and residual red blood cells were lysed using ACK lysing buffer (Lonza, MD, USA). The cells were treated with anti-CD16/32 antibody (BioLegend, Cat. No. 101302, CA, USA) for Fc-blocking before staining. Monoclonal murine-specific antibodies used in this study were selected according to the staining panels for each immune-cell subset. For analysis of M2 macrophages and ILC2s, cells were stained with a lineage cocktail consisting of biotin-conjugated anti-CD3, anti-Gr-1, anti-CD45R, and anti-Ter-119 antibodies (Cat. No. 133307, part 79751, 79750, 79752 and 79748) together with FITC-conjugated streptavidin (Cat. No. 405202), and further labeled with PE/Cy7-conjugated anti-CD127 (Cat. No.135014), PE-Dazzle 594-conjugated anti-CD206 (Cat. No. 141732), APC/Cy7-conjugated anti-F4/80 (Cat. No. 123118), AF700-conjugated anti-CD45 (Cat. No. 103128), BV650-conjugated anti-CD11b (Cat. No. 101239) (all from BioLegend), and AF647-conjugated anti-GATA3 antibody (BD Pharmingen, Cat. No. 560078, CA, USA). For NKT cell and Treg analysis, cells were stained with FITC-conjugated anti-CD3 (Cat. No. 553061), PerCP/Cy5.5-conjugated anti-CD4 (Cat. No. 100434), AF700-conjugated anti-CD45 (BioLegend), AF647-conjugated anti-Foxp3 (Cat. No. 560401) antibodies (BD Pharmingen), and T-Select mouse CD1d tetramer-PE (Medical & Biological Laboratories Co. Ltd., Tokyo, Japan). To stain transcription factors, intracellular staining was performed using a Transcriptional Factor Buffer Set according to the manufacturer’s instructions (Becton, Dickinson and Company, Franklin Lakes, NJ, USA). Dead cells were excluded using Zombie Aqua (BioLegend). Doublets were excluded based on forward scatter area versus height. The sequential gating strategy is provided in **Supplementary Figure 1**. CD45^+^ leukocytes were first identified, followed by lineage-negative cells and subsequent identification of ILC2s as Lin^-^CD127^+^GATA3^+^ cells. Fluorescence-minus-one (FMO) controls were not performed in this study; gating was based on our previously established and published flow cytometric protocol (5) and was applied consistently across all experimental groups. Fluorescence compensation was performed using single-stained compensation controls and verified before sample acquisition. Flow cytometry analyses were performed using an LSRFortessa™ flow cytometer (BD Biosciences). Instrument performance was verified according to the manufacturer’s standard quality-control procedures before each experiment. The data were analyzed using FlowJo software (Tree Star, OR, USA).

### Statistical calculations

Statistical analyses were performed using the GraphPad Prism 10 software program (GraphPad Software, MA, USA). Time-course experiments were analyzed using two-way analysis of variance (ANOVA) followed by Tukey’s multiple-comparisons test. All other comparisons were analyzed using one-way ANOVA followed by Tukey’s multiple-comparisons test. A P value < 0.05 was considered statistically significant. Data are presented as mean ± SD.

## Results

### αGalCer treatment exacerbates renal dysfunction and tubulointerstitial injury in adenine-induced CKD

**Figure 1:**
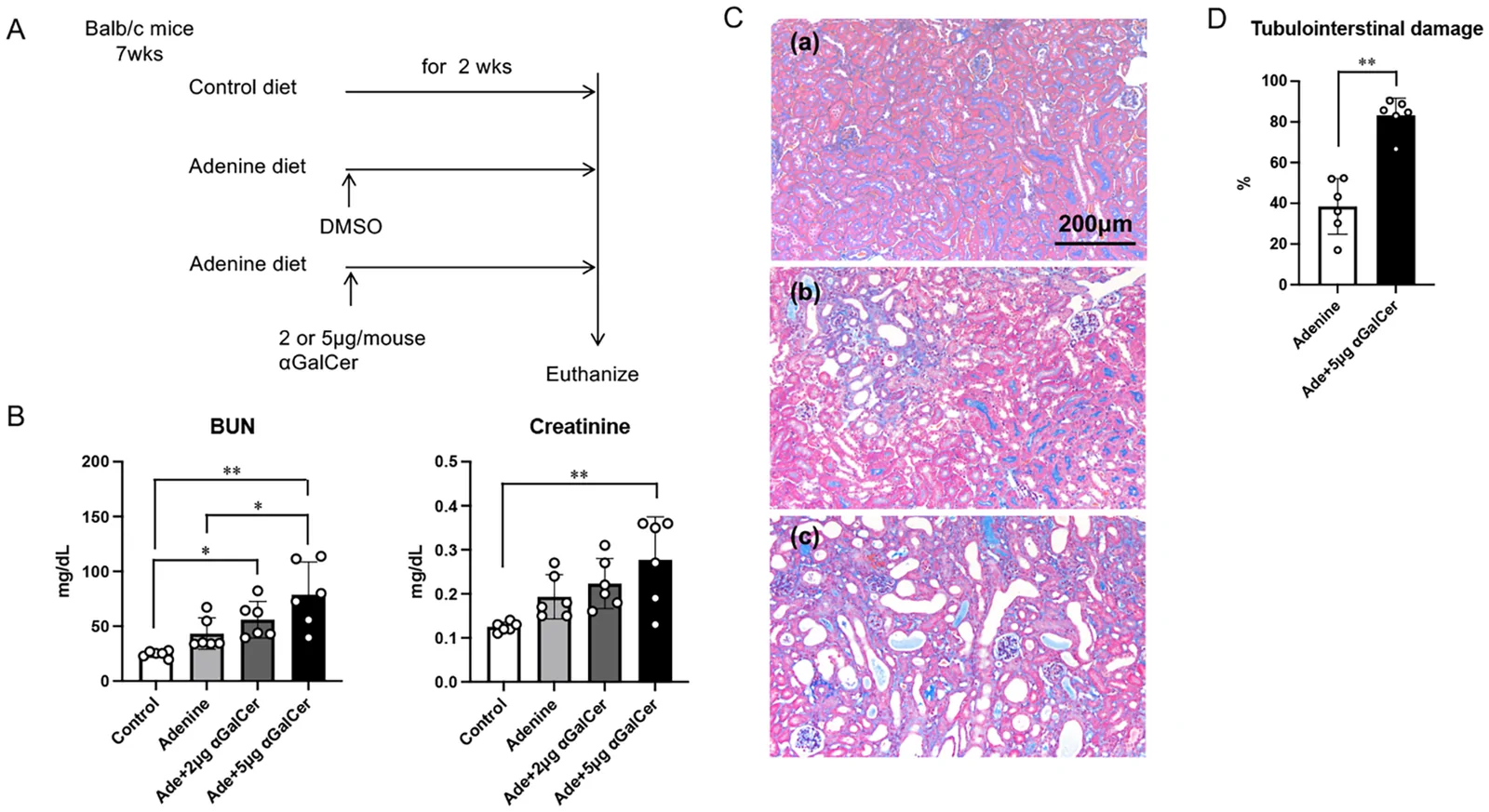
Effects of αGalCer treatment on renal dysfunction and tubulointerstitial injury in adenine-induced CKD. Male BALB/c mice were fed a control or adenine-containing diet for 2 weeks and administered vehicle or αGalCer. Renal function and histological injury were evaluated on day 14. **(A)** Experimental timeline. Seven-week-old male BALB/c mice received vehicle or αGalCer at 2 or 5 μg per mouse by intraperitoneal injection at the onset of diet feeding. **(B)** Serum blood urea nitrogen (BUN) and creatinine concentrations on day 14. The Y axes indicate serum BUN and creatinine levels. **(C)** Representative Masson’s trichrome–stained kidney sections: (a) control, (b) adenine + vehicle, and (c) adenine + αGalCer (scale bar = 200 μm; original magnification ×200). **(D)** Quantification of tubulointerstitial damage (%). The Y axis represents the percentage of the cortical area exhibiting tubular dilatation and interstitial tissue remodeling. Data are presented as mean ± SD. *P < 0.05, **P < 0.01. n = 6 mice per group.

To evaluate the impact of αGalCer treatment on adenine-induced CKD, male BALB/c mice were fed a 0.2% adenine diet for 2 weeks and administered αGalCer at doses of 2 or 5 μg per mouse. Adenine feeding induced renal dysfunction, as evidenced by increased blood urea nitrogen (BUN) and serum creatinine levels. Administration of αGalCer further exacerbated renal dysfunction in a dose-dependent manner, with the 5-μg dose showing the most pronounced effect (**Figures 1A, B**). Histological analysis using Masson’s trichrome staining demonstrated that αGalCer treatment aggravated tubulointerstitial injury, characterized by tubular dilatation and interstitial tissue remodeling. (**Figures 1C, D**). These results indicate that αGalCer treatment is associated with worsening of renal injury in adenine-induced CKD.

### αGalCer treatment enhances inflammatory and injury-related responses and shifts the renal immune milieu at late time points

**Figure 2:**
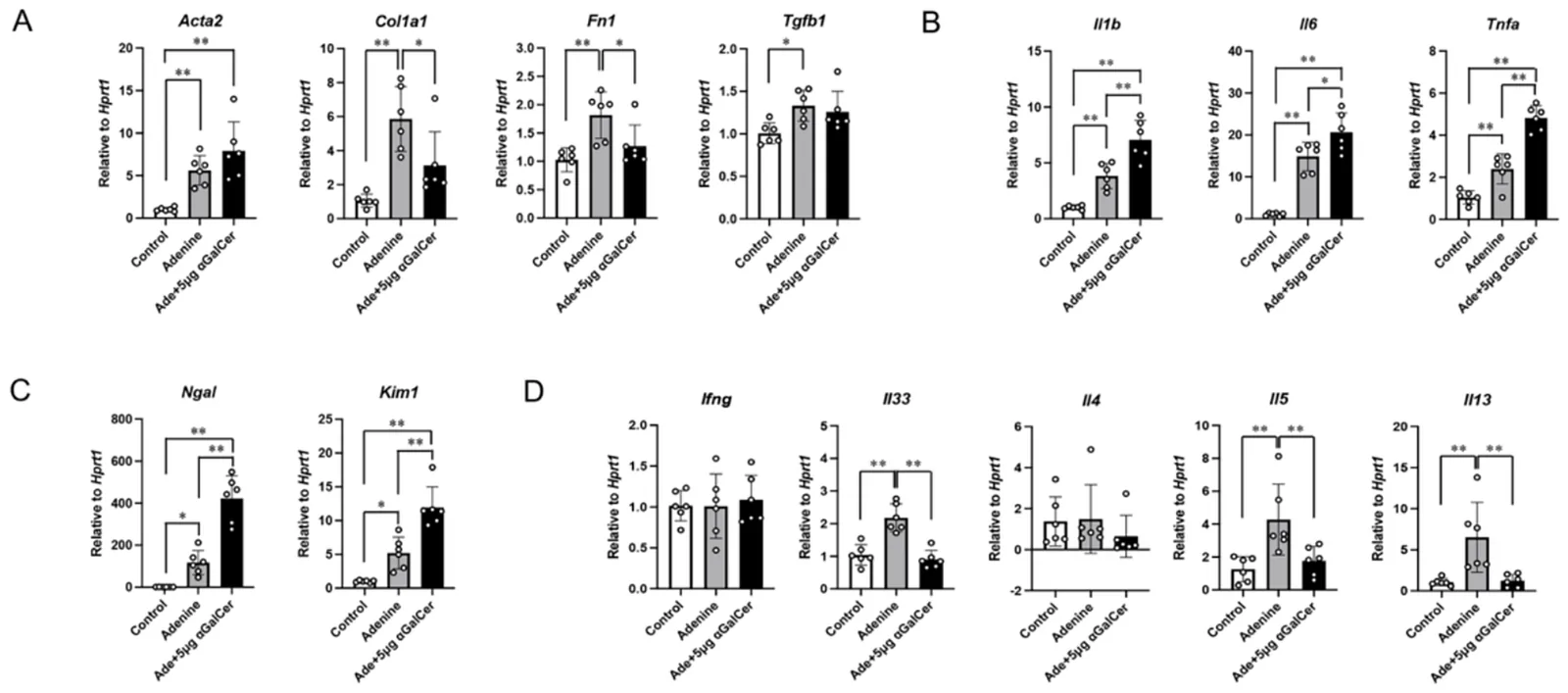
Renal gene expression profiles on day 14 in adenine-induced CKD with or without αGalCer treatment. Renal mRNA expression was analyzed by quantitative RT-PCR on day 14. Gene expression levels were normalized to *Hprt1* and are presented as relative expression levels. **(A)** Relative renal mRNA expression of fibrosis-related genes (*Acta2*, *Col1a1*, *Tgfb1*, *Fn1*). **(B)** Relative renal mRNA expression of inflammatory cytokines (*Il1b*, *Il6*, *Tnfa*). **(C)** Relative renal mRNA expression of tubular injury markers (*Lcn2*, *Havcr1*). **(D)** Relative renal mRNA expression of cytokines (*Ifng*, *Il33*, *Il4*, *Il5*, *Il13*). Data are presented as mean ± SD. *P < 0.05, **P < 0.01. n = 6 mice per group.

We next examined renal expression of genes associated with fibrosis, inflammation, tubular injury, and cytokine responses on day 14. Adenine feeding increased the expression of fibrosis-related genes, including *Acta2*, *Col1a1*, *Tgfb1*, and *Fn1*. However, αGalCer treatment did not further enhance these markers, and Col1a1 and Fn1 expression was significantly reduced compared with adenine alone (**Figure 2A**). These findings suggest that, at this early stage of adenine-induced CKD, αGalCer primarily augmented inflammatory and tubular injury responses rather than sustained fibrotic gene expression.

In contrast, αGalCer treatment markedly increased inflammatory and injury-related gene expression. The expression of *Il1b*, *Il6*, and *Tnfa* was significantly elevated, and tubular injury markers, including *Lcn2* (NGAL) and *Havcr1* (KIM-1), were also increased in αGalCer-treated mice (Adenine vs. Ade+5μg αGalCer, *Il1b*: p=0.0009, *Il6*: p=0.0226, *Tnfa*: p<0.0001, *Lcn2*: p<0.0001, *Havcr1*: p=0.0003, **Figures 2B, C**). Analysis of cytokine expression revealed suppression of type 2–associated cytokines, including *Il4*, *Il5*, and *Il13*, along with reduced *Il33* expression, whereas *Ifng* expression was not significantly altered at this time point (Adenine vs. Ade+5μg αGalCer, *Il5*: p=0.0206, *Il13*: p=0.0064, *Il33*: p<0.0001, **Figure 2D**). These findings indicate that αGalCer treatment is associated with enhanced inflammatory responses and a shift toward a type 1–dominant immune environment at later stages of CKD.

### αGalCer treatment induces early and transient IFN-γ expression accompanied by transient profibrotic gene responses

**Figure 3:**
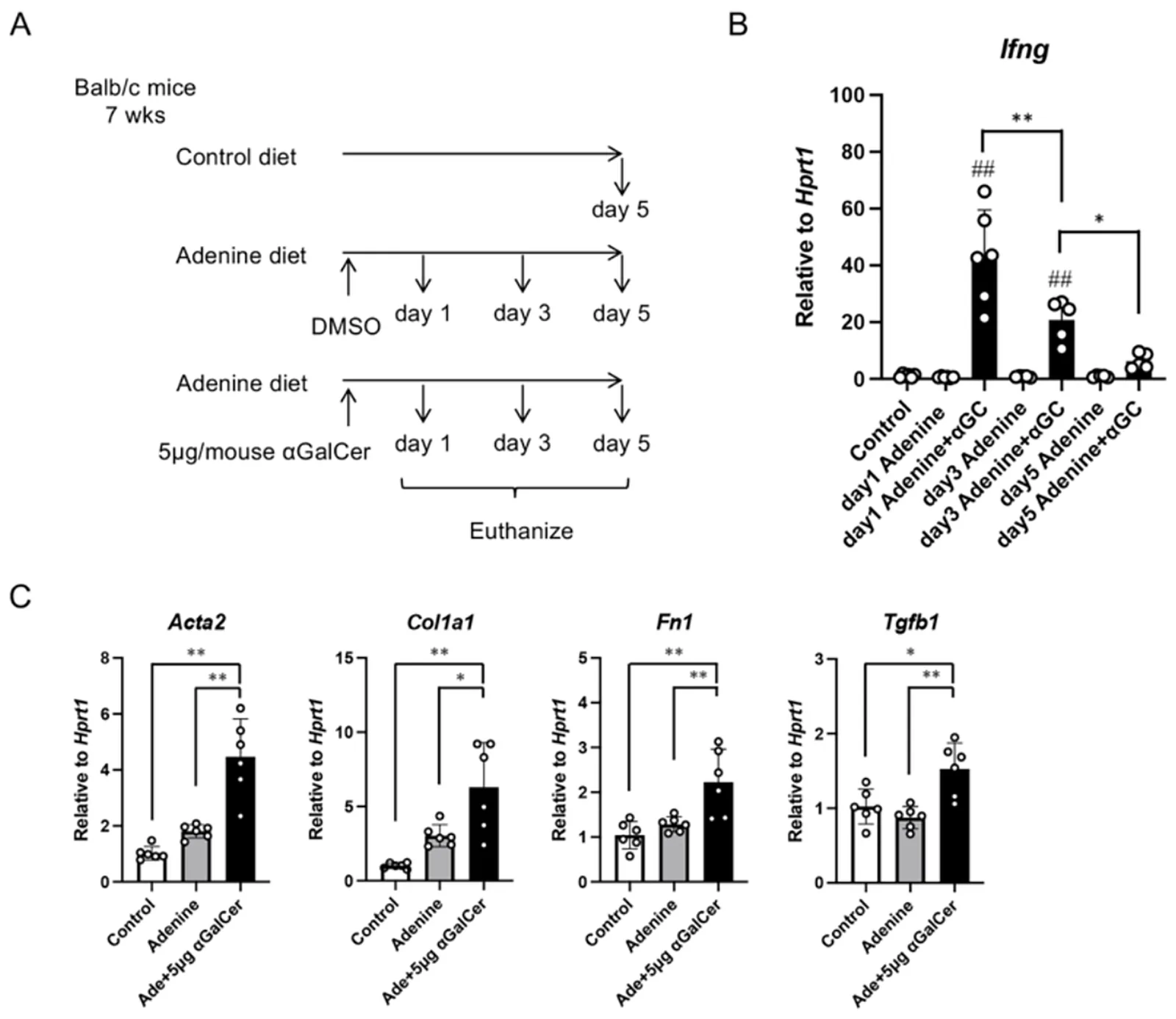
Temporal changes in renal *Ifng* and fibrosis-related gene expression following αGalCer treatment. Renal mRNA expression was analyzed by quantitative RT-PCR at the indicated time points after αGalCer administration. Gene expression levels were normalized to *Hprt1* and are presented as relative expression levels. **(A)** Experimental timeline. Mice were fed a control or adenine diet and administered αGalCer on day 0. Kidneys were collected at days 1, 3, and 5. **(B)** Relative renal *Ifng* mRNA expression at the indicated time points. **(C)** Relative renal mRNA expression of fibrosis-related genes (*Acta2*, *Col1a1*, *Tgfb1*, *Fn1*) on day 5. Data are presented as mean ± SD. *P < 0.05, **P < 0.01; #P < 0.05, ##P < 0.01 vs. control. n = 5–6 mice per group.

To investigate early immune responses following αGalCer administration, we performed a time-course analysis of renal *Ifng* expression. *Ifng* expression was rapidly induced and peaked at day 1 after αGalCer administration, followed by a decline toward baseline levels at later time points (**Figures 3A, B**).

We next examined gene expression at an early time point (day 5) to assess whether transient changes in fibrotic pathways were observed. At day 5, expression of fibrosis-related genes, including *Acta2*, *Col1a1*, *Tgfb1*, and *Fn1*, was significantly increased in the adenine + αGalCer group compared with adenine alone (Adenine vs. Ade+5μg αGalCer, *Acta2*: p=0.0001, *Col1a1*: p=0.00159, *Fn1*: p=0.0086, *Tgfb1*: p=0.0015, **Figure 3C**). These changes were not sustained at day 14, indicating a transient induction of fibrotic gene expression.

Together, these results demonstrate that αGalCer treatment is associated with an early and transient increase in *Ifng* expression, accompanied by transient activation of fibrosis-related transcriptional responses, followed by sustained inflammatory and injury-related changes at later stages.

### αGalCer treatment is associated with reduced renal ILC2 and Treg abundance, whereas no significant changes were observed in renal M2 macrophages or NKT cells

**Figure 4:**
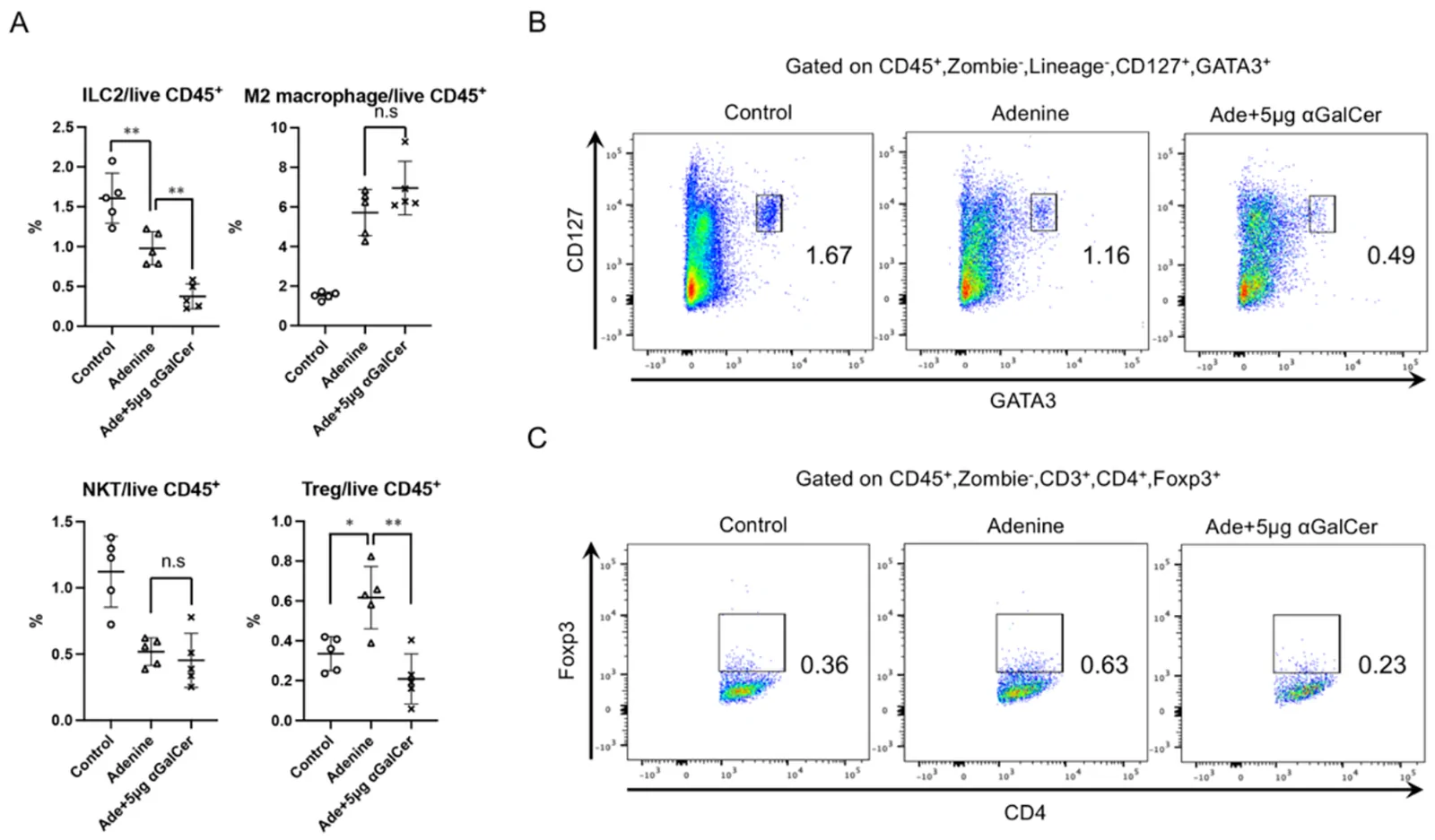
Effects of αGalCer treatment on renal immune-cell composition in adenine-induced CKD. Renal immune-cell subsets were analyzed by flow cytometry on day 14. Cell frequencies are expressed as the percentage of live CD45^+^ leukocytes. **(A)** Frequencies of renal ILC2s (Lin^-^CD127^+^GATA3^+^), M2 macrophages (F4/80^+^CD206^+^), NKT cells (CD3^+^CD1d–αGalCer tetramer^+^), and Tregs (CD4^+^Foxp3^+^). **(B)** Representative flow cytometry plots of ILC2s. **(C)** Representative flow cytometry plots of Tregs. Data are presented as mean ± SD. *P < 0.05, **P < 0.01; n.s., not significant. n = 5 mice per group.

We next examined the effects of αGalCer treatment on renal immune-cell composition. Flow cytometric analysis revealed that αGalCer administration significantly reduced the frequency of ILC2s (Lin^-^, CD127^+^, GATA3^+^) and regulatory T cells (CD4^+^, Foxp3^+^) among CD45^+^ kidney leukocytes compared with vehicle-treated controls (Adenine vs. Ade+αGC, ILC2s: p=0.0042, Treg: p=0.0007, **Figure 4A**). Representative gating plots confirmed the reduction in ILC2 and Treg populations (**Figures 4B, C**).

In contrast, the proportions of other immune populations, including M2 macrophages (F4/80^+^, CD206^+^) and NKT cells (CD3^+^, CD1d–αGalCer tetramer^+^), were not significantly altered (**Figure 4A**). These findings indicate that αGalCer treatment was associated with reductions in renal ILC2s and Tregs, whereas no significant changes were observed in renal M2 macrophages or NKT cells.

### IFN-γ neutralization ameliorates renal dysfunction associated with αGalCer treatment

**Figure 5:**
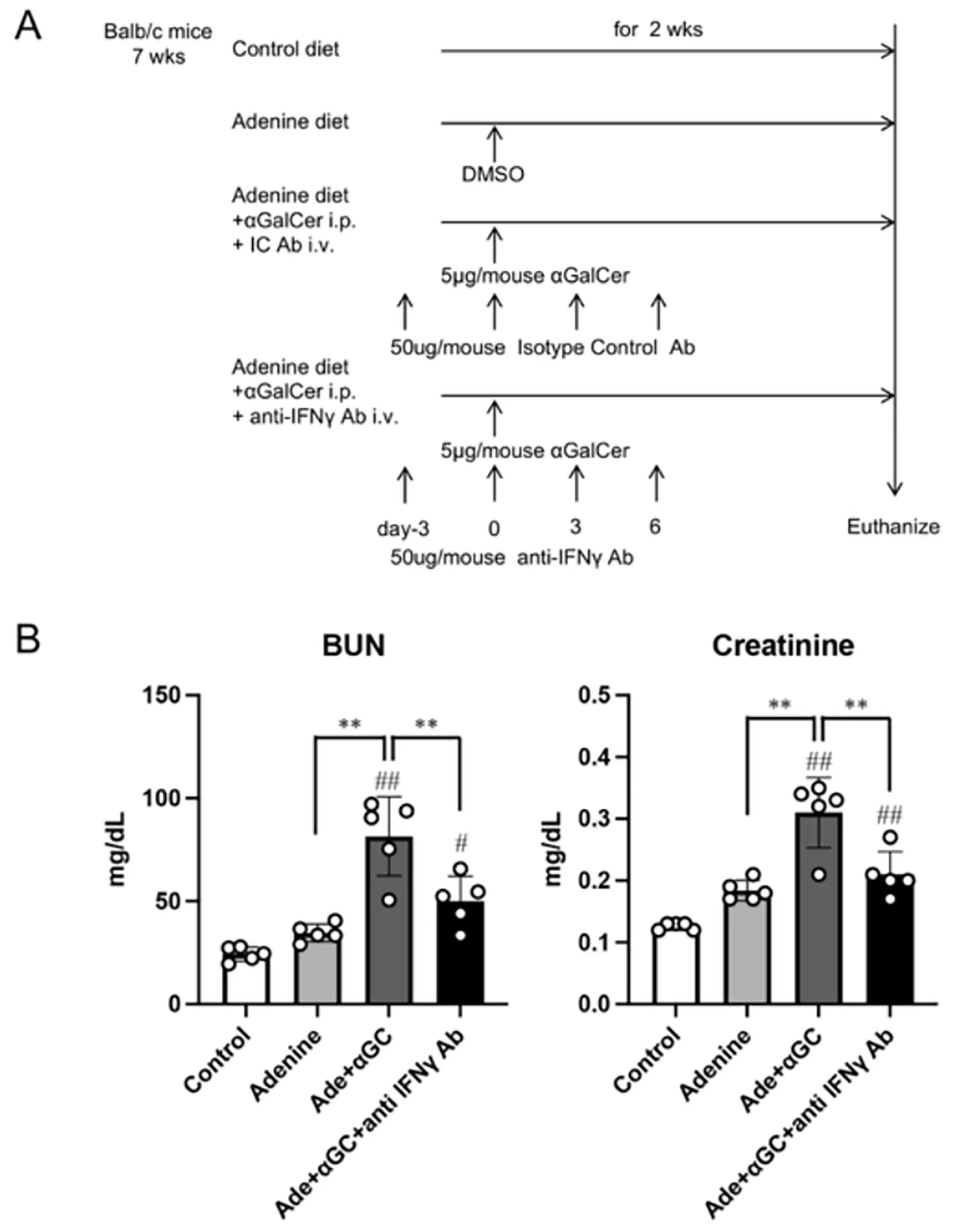
Effects of IFN-γ neutralization on renal dysfunction in αGalCer-treated mice. Male BALB/c mice fed an adenine-containing diet and treated with αGalCer received an anti–IFN-γ monoclonal antibody or isotype control, and renal function was evaluated on day 14. **(A)** Experimental timeline. **(B)** Serum blood urea nitrogen (BUN) and creatinine concentrations in the control, adenine, adenine + αGalCer, and adenine + αGalCer + anti–IFN-γ antibody groups. Data are presented as mean ± SD. **P < 0.01; #P < 0.05, ##P < 0.01 vs. control. n = 5 mice per group.

To assess the contribution of IFN-γ to the observed renal injury, mice were treated with an IFN-γ–neutralizing antibody during αGalCer administration. IFN-γ neutralization significantly reduced BUN and serum creatinine levels compared with αGalCer-treated mice receiving isotype control (Ade+αGC vs. Ade+αGC+anti-IFN-γ Ab, BUN: p=0.003, Creatinine: p=0.0018, **Figures 5A, B**). These findings indicate that IFN-γ is involved in the worsening of renal dysfunction associated with αGalCer treatment.

### IFN-γ neutralization attenuates tubular injury and reduces injury marker expression

**Figure 6:**
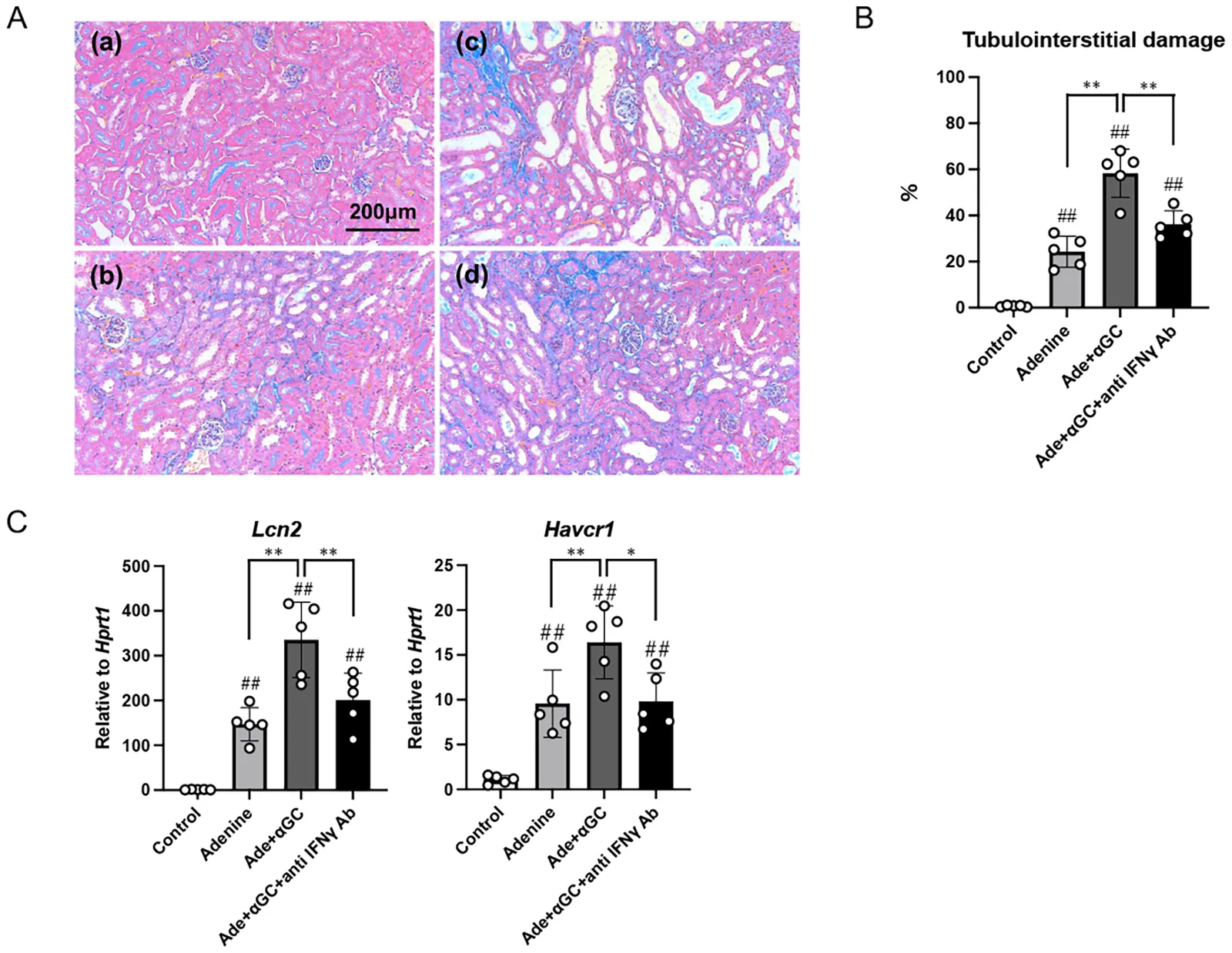
Effects of IFN-γ neutralization on tubular injury in αGalCer-treated mice. Male BALB/c mice were fed an adenine-containing diet, treated with αGalCer, and administered an anti–IFN-γ monoclonal antibody or isotype control. Histological injury and renal mRNA expression were evaluated on day 14. **(A)** Representative Masson’s trichrome–stained kidney sections: (a) control, (b) adenine, (c) adenine + αGalCer, (d) adenine + αGalCer + anti–IFN-γ antibody (scale bar = 200 μm). **(B)** Quantification of tubulointerstitial damage (%). The Y axis represents the percentage of the cortical area exhibiting tubular dilatation and interstitial tissue remodeling. **(C)** Relative renal mRNA expression of tubular injury markers (*Lcn2* and *Havcr1*). Gene expression levels were normalized to *Hprt1* and are presented as relative expression levels. Data are presented as mean ± SD. **P < 0.01; ##P < 0.01 vs. control. n = 5 mice per group.

Histological analysis demonstrated that IFN-γ neutralization markedly reduced tubulointerstitial injury compared with αGalCer-treated mice (Ade+αGC vs. Ade+αGC+anti-IFN-γ ab, p=0.0006, **Figures 6A, B**). Consistent with these findings, expression of tubular injury markers *Lcn2* and *Havcr1* was significantly decreased in mice receiving IFN-γ blockade (Ade+αGC vs. Ade+αGC+anti-IFN-γ ab, *Lcn2*: p=0.0068, *Havcr1*: p=0.0229, **Figure 6C**). These results indicate that IFN-γ contributes to tubular injury associated with αGalCer treatment.

### IFN-γ neutralization restores renal ILC2 abundance and partially alters immune-cell composition

**Figure 7:**
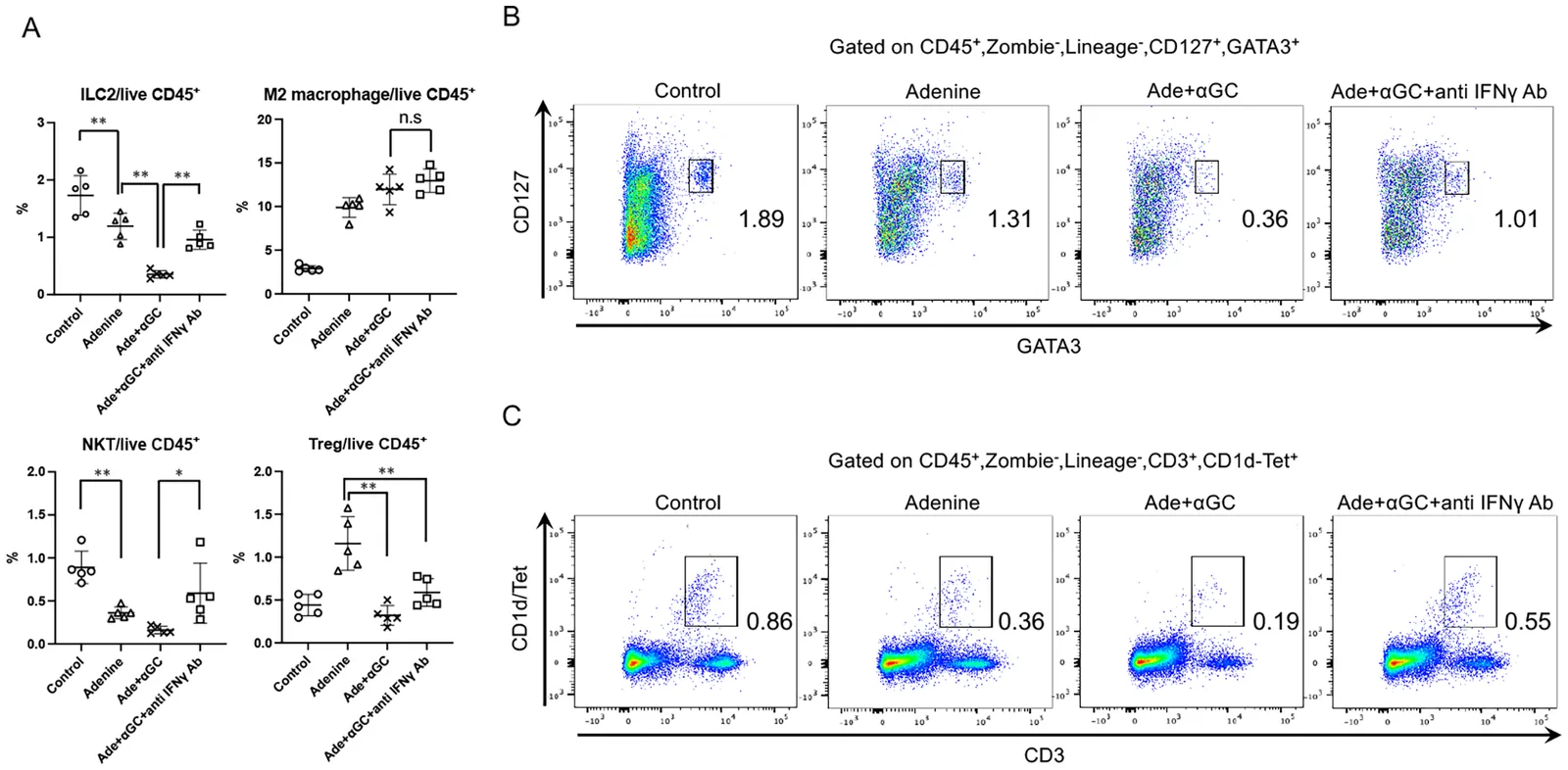
Effects of IFN-γ neutralization on renal immune-cell composition. Male BALB/c mice were fed an adenine-containing diet, treated with αGalCer, and administered an anti–IFN-γ monoclonal antibody or isotype control. Renal immune-cell subsets were analyzed by flow cytometry on day 14. Cell frequencies are expressed as the percentage of live CD45^+^ leukocytes. **(A)** Frequencies of renal ILC2s (Lin^-^CD127^+^GATA3^+^), M2 macrophages (F4/80^+^CD206^+^), NKT cells (CD3^+^CD1d–αGalCer tetramer^+^), and Tregs (CD4^+^Foxp3^+^). **(B)** Representative flow cytometry plots of ILC2s. **(C)** Representative flow cytometry plots of NKT cells. Data are presented as mean ± SD. **P < 0.01; n.s., not significant. n = 5 mice per group.

Finally, we evaluated the effects of IFN-γ neutralization on renal immune-cell populations. IFN-γ blockade significantly increased the frequency of renal ILC2s compared with αGalCer-treated controls (Ade+αGC vs. Ade+αGC+anti-IFN-γ ab, ILC2s: p=0.0033, **Figure 7A**). In contrast, Treg frequencies showed limited recovery, and M2 macrophage proportions remained unchanged across groups.

Interestingly, IFN-γ neutralization was associated with an increase in renal NKT-cell frequency. Representative flow cytometry plots confirmed these changes (Ade+αGC vs. Ade+αGC+anti-IFN-γ ab, NKT cells: p=0.0192, **Figures 7B, C**). These findings indicate that IFN-γ plays a key role in shaping renal immune composition, particularly in regulating ILC2 abundance, in the context of αGalCer treatment.

## Discussion

CKD progression reflects a complex interplay between epithelial injury, inflammation, and maladaptive repair, ultimately leading to tubulointerstitial fibrosis. While numerous immune pathways have been implicated in this process, the upstream mechanisms that disrupt reparative immune responses remain incompletely defined. In the present study, we demonstrate that an IFN-γ–dominant immune environment is associated with exacerbation of kidney injury, suppression of renal ILC2s, and impaired tissue repair in a murine model of adenine-induced CKD.

ILC2s have emerged as key regulators of tissue protection and repair in the kidney (3, 4). Recent studies have further highlighted the diverse reparative functions of tissue-resident ILC2s in kidney diseases, such as amphiregulin-mediated tissue repair and maintenance of local immune homeostasis (21, 22). These cells respond to alarmins such as IL-33 and produce amphiregulin and type 2 cytokines that promote epithelial integrity, modulate macrophage polarization, and restrain fibrotic remodeling (3–6). Consistent with previous reports, we observed a marked reduction in renal ILC2s during CKD progression (5, 6). Our findings further suggest that this reduction is associated with a shift toward a type 1–dominant immune environment, characterized by increased IFN-γ signaling and suppression of type 2 cytokines. These observations support the concept that disruption of the balance between type 1 and type 2 immunity may represent a critical determinant of disease progression in CKD.

IFN-γ is a well-established negative regulator of ILC2 function. Previous studies have shown that IFN-γ signaling suppresses ILC2 proliferation, cytokine production, and responsiveness to IL-33 (7, 8, 23). In line with these findings, we observed that IFN-γ neutralization restored renal ILC2 abundance and ameliorated kidney injury in the setting of αGalCer administration. Together with these previous studies, our findings support the concept that an IFN-γ–dominant immune environment is associated with reduced renal ILC2 responses during CKD progression.

In this study, αGalCer was used as a tool to induce a type 1–skewed immune environment. αGalCer is widely used to activate invariant NKT cells (11, 24); however, it may also influence other immune populations indirectly through cytokine networks. Therefore, although NKT cells are a likely early source of IFN-γ following αGalCer administration, we did not directly demonstrate that NKT-derived IFN-γ is solely responsible for the observed effects. Rather, our findings support the interpretation that αGalCer-induced immune activation results in a transient IFN-γ surge that shifts the renal immune balance toward a type 1–dominant state.

Time-course analysis revealed that *Ifng* expression was rapidly and transiently induced following αGalCer administration, preceding sustained inflammatory and injury responses. This temporal pattern suggests that early cytokine signals may initiate downstream immune and tissue responses that persist beyond the initial IFN-γ surge. Given the sensitivity of ILC2s to IFN-γ–mediated suppression (7, 25), even transient exposure to elevated IFN-γ may have lasting consequences on their maintenance and function. Because the present study employed a 2-week adenine model, which represents an early stage of CKD characterized by ongoing inflammation and tissue remodeling, these transcriptional changes should be interpreted as transient profibrotic responses rather than evidence of established fibrosis. This interpretation is consistent with previous characterizations of the adenine-induced CKD model, in which early inflammatory responses precede established collagen deposition (20). We also state that longer-term studies will be required to determine whether these early transcriptional changes ultimately lead to progressive renal fibrosis.

Interestingly, although both ILC2s and Tregs were reduced following αGalCer treatment, restoration of ILC2s by IFN-γ neutralization was more pronounced than that of Tregs. This observation suggests that ILC2s may be particularly sensitive to IFN-γ–mediated regulation in the kidney. In addition, despite reductions in Tregs, renal injury was exacerbated, supporting the notion that loss of ILC2-mediated epithelial protection may play a dominant role in this model of CKD.

The IL-33–ILC2 axis represents a key upstream pathway in renal type 2 immunity (3, 5, 6). In the present study, expression of *Il33* was reduced following αGalCer treatment, suggesting that IFN-γ–dominant immune conditions may disrupt not only ILC2 abundance but also upstream epithelial–immune signaling. This dual effect may further amplify impairment of reparative responses.

Our findings also highlight the importance of immune balance rather than individual cell types in determining disease outcomes. While previous studies have demonstrated both protective and pathogenic roles for NKT cells depending on context (26, 27), our results suggest that excessive immune activation leading to IFN-γ dominance may override reparative pathways and promote tissue injury.

This study has several limitations. First, the adenine-induced CKD model primarily represents crystal-associated tubulointerstitial nephropathy and does not fully recapitulate the diverse etiologies of human CKD, such as diabetic kidney disease or hypertensive nephrosclerosis. Furthermore, αGalCer-induced immune activation does not fully recapitulate physiological immune responses in CKD, and the specific cellular sources of IFN-γ were not directly identified. In addition, direct activation of IFN-γ signaling in renal ILC2s (e.g., STAT1 activation or GATA3 suppression) was not examined in the present study. Although previous studies have established that IFN-γ suppresses ILC2 function through STAT1 activation and subsequent inhibition of GATA3 (7, 8), whether this mechanism operates in the present CKD model warrants further investigation. Second, the causal role of ILC2 depletion in disease progression was not directly tested, and other immune populations may contribute to the observed phenotype (28). Third, IFN-γ neutralization was evaluated only in the context of αGalCer treatment, and its effects in baseline CKD without immune stimulation remain to be determined. Studies using adenine-fed IFN-γ-deficient mice and IFN-γ blockade in the absence of αGalCer are currently underway to address this important question. Fourth, renal ILC2s were identified as Lin^-^CD127^+^GATA3^+^ cells. Although this gating strategy has been used in our previous studies, additional phenotypic markers, functional analyses, and absolute quantification of renal ILC2s would provide a more comprehensive characterization of these cells and should be addressed in future studies. Fifth, cytokine expression was primarily evaluated at the mRNA level in whole-kidney tissue. Although complementary protein-level analyses would provide additional information, the present study focused on characterizing changes in the local renal immune environment using multiple independent approaches, including renal function, histopathology, flow cytometry, and quantitative RT-PCR. Finally, only male mice were used in this study; therefore, future studies should determine whether similar findings are observed in female mice. Future studies using single-cell transcriptomic approaches may further define the cellular sources of IFN-γ and clarify how immune-cell interactions regulate reparative ILC2 responses during CKD progression (22).

Despite these limitations, our study provides evidence that IFN-γ–dominant immune polarization is associated with suppression of protective ILC2 responses and exacerbation of CKD. These findings support a model in which disruption of the balance between type 1 and type 2 immunity contributes to impaired tissue repair and disease progression. Therapeutic strategies aimed at modulating IFN-γ signaling or preserving ILC2 function may therefore represent promising approaches to mitigate chronic kidney injury.

In summary, our findings support the concept that an IFN-γ–dominant immune environment is associated with reduced renal ILC2s and worsened kidney injury in CKD. Restoration of immune balance through IFN-γ neutralization partially rescues ILC2 abundance and improves disease outcomes. These findings highlight immune imbalance as a key feature of CKD progression and provide a framework for targeting reparative immune pathways in CKD.

## Data Availability

The original contributions presented in the study are included in the article/**Supplementary Material**. Further inquiries can be directed to the corresponding author.
