# Supplementary material for: IFN-γ–dominant immune shift is associated with reduced protective ILC2 responses in chronic kidney disease
Source: Front Immunol. 2026 Sep 8;17:1870937. doi: 10.3389/fimmu.2026.1870937 (PMC13593754; doi:10.3389/fimmu.2026.1870937)
Supplement: Supplementary file 1 [file DataSheet1.pdf]

Supplementary Figure S1. Gating strategy for the identification of renal ILC2s by flow cytometry.

Representative gating strategy used to identify renal ILC2s from kidney single-cell suspensions. ILC2s were defined as $Lin^{-}CD127^{+}GATA3^{+}$ cells after sequential gating on lymphocytes, singlets, live $CD45^{+}$ leukocytes, and lineage-negative cells.

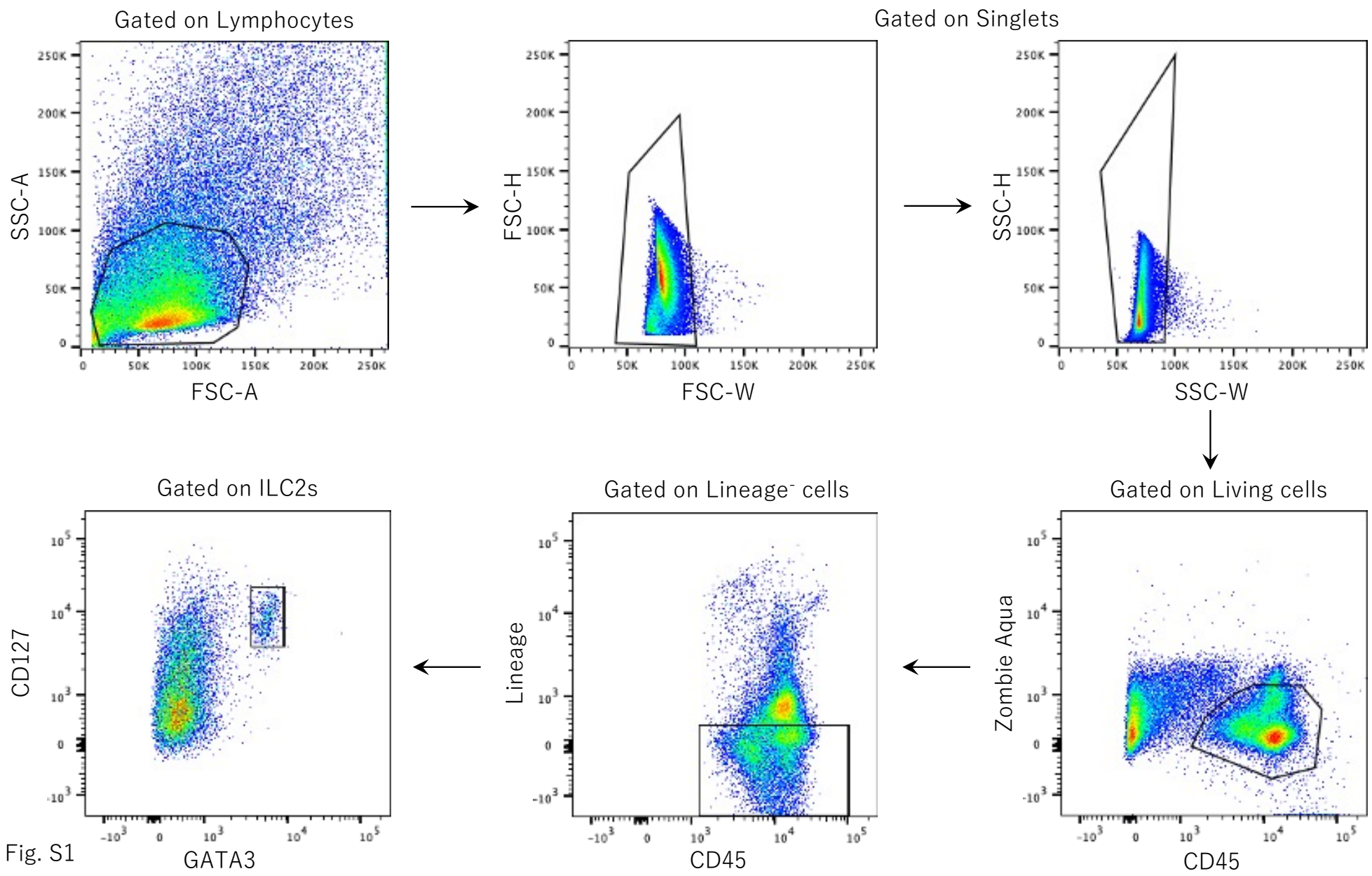

Supplementary Fig. S1
